# Phytochemical and Functional Diversity of Enzyme-Assisted Extracts from *Hippophae rhamnoides* L., *Aralia cordata* Thunb., and *Cannabis sativa* L.

**DOI:** 10.3390/antiox13080950

**Published:** 2024-08-05

**Authors:** Viktorija Januskevice, Ana Maria Gomes, Sérgio Sousa, Joana Cristina Barbosa, Rita Vedor, Paulina Martusevice, Mindaugas Liaudanskas, Vaidotas Zvikas, Pranas Viskelis, Laima Cesoniene, Aiste Balciunaitiene, Jonas Viskelis, Sonata Szonn, Dalia Urbonaviciene

**Affiliations:** 1 Institute of Horticulture, Lithuanian Research Centre for Agriculture and Forestry, 54333 Kaunas, Lithuania; paulina.martusevice@lammc.lt (P.M.); aiste.balciunaitiene@lammc.lt (A.B.); jonas.viskelis@lammc.lt (J.V.); dalia.urbonaviciene@lammc.lt (D.U.); 2 CBQF—Centro de Biotecnologia e Química Fina—Laboratório Associado, Escola Superior de Biotecnologia, Universidade Católica Portuguesa, Rua Diogo Botelho 1327, 4169-005 Porto, Portugal; amgomes@ucp.pt (A.M.G.); sdsousa@ucp.pt (S.S.); jcbarbosa@ucp.pt (J.C.B.); s-rcvedor@ucp.pt (R.V.); 3 Botanical Garden, Vytautas Magnus University, Z.E. Zilibero 6, 46324 Kaunas, Lithuania; laima.cesoniene@vdu.lt; 4 Institute of Pharmaceutical Technologies, Lithuanian University of Health Sciences, LT-50166 Kaunas, Lithuania; mindaugas.liaudanskas@lsmu.lt (M.L.); vaidotas.zvikas@lsmu.lt (V.Z.); sonata.szonn@lsmu.lt (S.S.); 5 Research Institute of Natural and Technological Sciences, Vytautas Magnus University, 40444 Kaunas, Lithuania

**Keywords:** *Aralia cordata*, sea buckthorn, industrial hemp, leaves, root, enzyme-assisted extraction, functional properties

## Abstract

Plant leaves are a source of essential phenolic compounds, which have numerous health benefits and can be used in multiple applications. While various techniques are available for recovering bioactive compounds from by-products, more data are needed on enzyme-assisted extraction (EAE). The aim of this study was to compare EAE and solid–liquid extraction (SLE), to evaluate the impact on bioactive compounds’ extraction yield, phytochemical composition, and the antioxidant, antimicrobial, and antidiabetic properties of *Aralia cordata* leaves and roots, sea buckthorn *Hippophae rhamnoides,* and hemp *Cannabis sativa* leaves. The results indicate that EAE with Viscozyme L enzyme (EAE_Visc) extracts of the tested plant leaves possess the highest yield, antioxidant activity, and total phenolic content. Moreover, the EAE_Visc extract increased by 40% the total sugar content compared to the control extract of *A. cordata* root. Interestingly, the sea buckthorn leaf extracts exhibited α-glucosidase inhibitory activity, which reached an almost 99% inhibition in all extracts. Furthermore, the sea buckthorn leaves SLE and EAE_Visc extracts possess antibacterial activity against *Staphylococcus aureus*. Additionally, scanning electron microscopy was used to examine changes in cell wall morphology after EAE. Overall, this study shows that EAE can be a promising method for increasing the yield and improving the functional properties of the resulting extracts in a fast and sustainable way compared to SLE.

## 1. Introduction

Growing interest has been observed in exploring natural sources’ phytochemical composition and functional properties in recent years [1]. The by-products of agro-industries, such as plant leaves, roots, and stems, are emerging as promising renewable resources rich in cellulose, hemicellulose, and lignin [2]. Their complex matrix traps and binds bioactive substances such as phenolic compounds, amino acids, proteins, minerals, and lipids, which offer numerous health benefits and have potential applications in various industries, in particular in the food and food supplement industries [3]. Moreover, consumers’ awareness of diet and health correlations is rising steadily [4]. If prepared appropriately, plant by-products can deliver prebiotic, anti-diabetic, and antioxidant functions. For example, hydrolytic treatment has enabled the extraction of previously non-extractable polyphenols, resulting in higher phenolic recovery in plant-by product extracts [5]. However, different plant materials and their morphological parts possess miscellaneous compositions, which can fluctuate positively and negatively, accentuating the need for a fundamental analysis. Furthermore, the rising interest in by-product development is fully aligned with the United Nations Sustainable Development Goals (SDGs)—in particular, SDG No. 12, Responsible Consumption and Production, which aims to cut food loss and waste in food production by up to 50%, and SDG, No. 2, End Hunger, achieving food security and improved nutrition, and promoting sustainable agriculture [6]. Following these rationales, investigating the possible functionality of plants is crucial, and sea buckthorn (*Hippophae rhamnoides* L.), *Aralia cordata* Thunb. (*A. cordata*), and industrial hemp (*Cannabis sativa* L.) are plant species that have gained attention due to their rich phytochemical composition and promising functional properties [7,8,9,10,11]. 

The sea buckthorn (*H. rhamnoides*) is a valuable plant known for its rich phytochemical composition in both its berries and leaves. The berries contain essential compounds such as carotenoids, flavonoids, phenolic acids, fatty acids, ascorbic acid, and tocopherols, contributing to their powerful antioxidant, anti-inflammatory, and anti-cancer properties. Additionally, the leaves are rich in flavonols (rutin, Q-3-*O*-galactoside, I-3-*O*-glucoside, K-3-*O*-glucoside, K-3-*O*-rutinoside, quercetin, and kaempferol), phenolic acids (gallic acid, caffeic acid, *p*-coumaric acid, ferulic acid) vitamins, proteins, and minerals [12,13], which have been found to have anti-obesity, hypoglycaemic, antioxidant, antibacterial, anti-inflammatory, antidiabetic and anti-cardiovascular disease properties [14,15]. While the berries are commonly used for their nutritional and medicinal benefits, the leaves, utilised in herbal preparations, also possess significant therapeutic potential. 

Meanwhile, *A. cordata* is a plant that is highly valuable due to its rich content of bioactive compounds such as polyphenols, anthocyanins, carbohydrates, and vitamins [7,16]. These compounds have been found to possess various pharmacological properties, including anti-inflammatory, cardiovascular, and nervous system protection, compound metabolism regulation, and antibacterial, antiviral, and antioxidant properties. Moreover, the plant has been traditionally used to treat ailments such as hepatitis and rheumatoid arthritis. In a previous study, *A. cordata* roots and leaves were found to possess significant antioxidant activity and total phenolic content [7]. 

Hemp (*C. sativa* L.) is a widespread plant species of the *Cannabaceae* family found in different habitats. The interest in hemp has remarkably increased due to specific phytochemicals in its leafy anatomical parts. More than 70 biologically active and unique to Cannabis terpenophenolic compounds, phytocannabinoids, have been found [17]. Several research studies showed the health-promoting and medicinal properties of phytocannabinoids. Among them, Δ^9^-tetrahydrocannabinol (Δ^9^-THC) is a well-known natural psychotropic compound; for this reason, only the approved cultivars of *C. sativa* accumulating less than 0.2–0.3% of Δ^9^-THC are officially allowed in Canada, the USA, and many European countries. Hemp leaves are rich in phytochemicals, like cannabinoids, terpenes, phenolic compounds, and their biosynthetic routes. Cannabinoids represent the most studied group of compounds, mainly due to their wide range of pharmaceutical effects in humans, including psychotropic activities [18]. 

Human health, from a holistic viewpoint, requires a comprehensive approach. A highly balanced and functional nutrition has to deliver a whole spectrum of particular properties such as antioxidant, antidiabetic, and antimicrobial ones, among others, together with the nutritional value of the food. Hence, products with a high nutritional value can be enriched with plant by-products [19]. Recently, the antidiabetic activity of extracts has been garnering attention. This activity can also be associated with obesity prevention. More than 650 million adults and 340 million children suffer from obesity, and food is one of the key factors in preventing it [20]. For example, the study of berry pomace extracts showed a high α-glucosidase and pancreatic lipase inhibition capacity, which are referred to as antidiabetic properties [21]. 

In order to isolate bioactive compounds and guarantee product quality, efficacy, and safety, the extraction process is essential in the food and pharmaceutical industries. Conventional extraction methods, such as soxhlet extraction, solid–liquid extraction, and maceration, involve subjecting the raw material to high temperatures with chemical solvents. While this practice enhances the yield of compounds, it concurrently diminishes the quality and degrades valuable compounds, due to prolonged exposure and considerable energy consumption [22,23]. Lately, innovative technologies, also known as “green technologies”, have been developed for extracting bioactive compounds without using toxic chemicals [24]. These eco-friendly techniques are directly related to the environment and consumer health [25]. Notable innovative methods for extracting bioactive compounds include supercritical fluid, microwave-assisted, enzyme-assisted, ultrasound-assisted, pressurised liquid, and pulsed electric field techniques [26]. Enzyme-assisted extraction has been observed to offer multiple benefits compared to conventional extraction techniques, due to its eco-friendly nature and improved ability to extract specific compounds under gentle processing conditions, shorter extraction times, and reduced solvent, as well as energy, usage [27]. Additionally, various factors, including the type and concentration of enzymes, extraction duration, substrate-to-solvent ratio, pH, and temperature, should be considered to obtain extracts with higher yields and improved properties [28].

**Figure 1:**
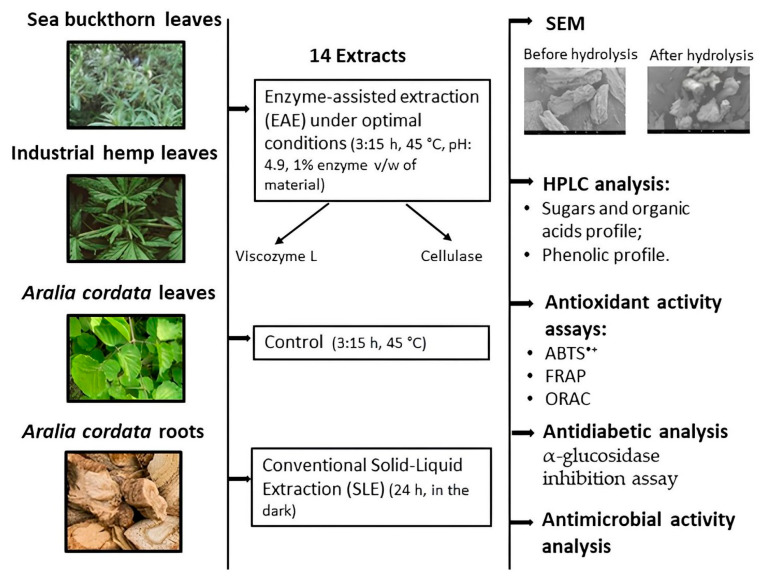
Overview of the experimental design.

The aim of this research is to evaluate and compare sea buckthorn leaf, industrial hemp leaf, and *A. cordata* leaf and root extracts obtained by different methods, namely, enzyme-assisted extraction (EAE) and solid–liquid extraction (SLE). This study covers the selection of the raw material and the preparation, extraction, and evaluation of phytochemical composition to achieve a high yield of biologically active substances and effective antioxidant, antidiabetic, and antimicrobial activities. Figure 1 provides an overview of the overall experimental design for the production and characterization of a total of 14 extracts.

## 2. Materials and Methods

### 2.1. Plant Material

Sea buckthorn (*Hippophae rhamnoides* L.) and *Aralia cordata* plants were grown at the Institute of Horticulture, Lithuanian Research Centre of Agriculture and Forestry experimental fields (55°08′ N, 23°80′ E). Industrial hemp (*Canabis sativa* L.) leaves were obtained from a local food factory, “Allive Europe” (Voskoniai, Lithuania). Fresh *A. cordata*, sea buckthorn, and industrial hemp leaves were harvested in 2021. The collected leaves and roots were instantly frozen at −35 °C before freeze-drying. The samples were lyophilised in Zirbus lyophiliser (Zirbus Technology GmbH, Bad Grund, Germany) at 0.01 mbar pressure and −85 °C condenser temperature. The freeze-dried samples were ground to a powder (particle size 0.2 mm) using a Retsch 200 knife mill (Haan, Germany) and stored in a sealed container before the analysis.

### 2.2. Enzyme Products

Cellulase and Viscozyme L were purchased from Sigma-Aldrich (Steinheim, Germany). Cellulase was produced from *Trichoderma reesei* and declared to have ≥700 units/g. Viscozyme L is a cellulolytic enzyme complex from *Aspergillus aculeatus*. Furthermore, the manufacturer states that the enzyme mixture contains a wide range of arabanase, Cellulase, β-glucanase, hemicellulase, and xylanase. The product is declared to have ≥100 FBGU/g. 

### 2.3. Enzyme-Assisted Extraction (EAE) and Solid-Liquid Extraction (SLE)

Enzyme-assisted extraction (EAE) was carried out as outlined by Puzeryte et al. under optimal conditions [8]. *Aralia cordata* leaves and roots, sea buckthorn leaves, and industrial hemp leaves were extracted using EAE at a 1:20 (*w*/*v*) ratio and mixed to obtain a homogeneous suspension. The suspension pH, 4.9, was adjusted using 6 M HCl and 0.5 M NaOH, and the enzymes Viscozyme L and Cellulase 1% (*v*/*w* of dry matter) were added. EAE was carried out at 45 °C in an incubator for 3:15 h. After extraction, the enzyme was deactivated by heating the hydrolysed material at 95 °C for 10 min. Then, the separation of the suspension into liquid and solid fractions was accomplished using a filter (200 mesh). Sample extracts (liquid fraction) were frozen and stored at −35 °C before analysis. Furthermore, the extract to be used as a control was prepared under optimal conditions without enzyme addition. SLE of the tested plant sources was performed in a ratio of 1:20 (*w*/*v*) with H_2_O for 24 h in the dark. All the experiments were performed in triplicate.

### 2.4. Protein Content by Lowry Assay

The protein content of the sample extracts was determined using the colorimetric method described by Lowry et al. [29].

### 2.5. Determination of Sugars and Organic Acids

Sugars and organic acid concentrations were determined through high-performance liquid chromatography (HPLC). Briefly, the samples were centrifuged and the supernatants were collected, filtered through 0.22 µm filters, and injected into the HPLC system for analysis. The HPLC system consisted of an Aminex HPX-87H cation exchange column (300 × 7.8 mm) (Bio Rad Laboratories Pty Ltd., CA, USA), coupled to refractive index and ultra-violet detectors. Throughout the analysis, the column was maintained at 40 °C, and the mobile phase utilised was 5 mM H_2_SO_4_ at a flow rate of 0.7 mL min^−1^. The compounds were identified by the retention time and quantified through the area of the corresponding peak by interpolation of calibration curves determined from the respective standards. 

### 2.6. Determination of Total Phenolic Content

The total polyphenol content (TPC) in the extracts was determined according to the Folin–Ciocalteu method [30], using gallic acid (GA) as the standard, according to the method of Bobinaite et al. [31]. The reagent was prepared by diluting a stock solution with ultra-pure distilled water (1/10, *v*/*v*). The samples (1.0 mL, three replicates) were introduced into test cuvettes, followed by 5.0 mL of Folin–Ciocalteu’s phenol reagent and 4.0 mL of Na_2_CO_3_ (7.5%). The system was then placed at ambient temperature for 1 h. The absorbance of all the samples was measured at 765 nm using a Cintra 202 (“GBC Scientific Equipment”, Knox, Australia) spectrophotometer. The total concentration of phenolic compounds was determined from the calibration curve and expressed in mg of gallic acid equivalents in 100 mL of extract. 

### 2.7. Evaluation of Phenolic Compounds in Extracts Using the UHPLC-ESI-MS/MS Technique

The qualitative and quantitative content of phenolic compounds in the tested *A. cordata* leaf and root, sea buckthorn leaf, and industrial hemp leaf samples extract were evaluated using ultra-high-performance liquid chromatography (UHPLC) coupled to a mass spectrometer. The method employed was described and validated in an article by Gonzalez-Burgos et al. [32]. The phenolic content of the sample extract was analysed using a liquid chromatography system, “Waters ACQUITY UPLC^®^ H–Class”, with a tandem quadrupole mass detector, “Xevo TQD” (Waters, Milford, MA, USA). The compounds were separated using a “YMC Triart C18” (100 Å, 100 × 2.0 mm; particle size 1.9 μm) with a pre-column. Both qualitative and quantitative analyses were performed. The mass spectrometry parameters for the phenolic compound analysis are presented in Table 1.

### 2.8. Determination of Antioxidant Activity

An ABTS^•+^ radical cation decolourization assay was applied according to the methodology described by Re et al. [33]. An amount of 2 mL of ABTS^•+^ (2,2′-azino-bis(3-ethylbenzthiazoline-6-sulphonic acid)) solution, with an absorbance of 0.800 ± 0.02, was mixed with 20 μL of the samples. After 30 min, the absorbance of each sample was measured at 734 nm using a Cintra 202 spectrophotometer (GBC Scientific Equipment, Knox, Braeside, VIC, Australia) to determine the decrease in absorbance.

A ferric ion reducing antioxidant power (FRAP) assay was conducted following the method described by Benzie and Strain [34], with some modifications. The FRAP solution was made by combining TPTZ (0.01 M dissolved in 0.04 M HCl), FeCl_3_ × 6H_2_O (0.02 M in water), and acetate buffer (0.3 M, pH 3.6) at a ratio of 1:1:10. A quantity of 2 mL of the freshly prepared FRAP reagent was mixed with 20 μL of the samples. After 30 min, the absorbance increase was determined at 593 nm using a Cintra 202 (GBC Scientific Equipment, Knox, Australia) spectrophotometer.

All antioxidant activity assays were calculated using Trolox calibration curves and expressed as the μmol of the Trolox equivalent (TE) per one millilitre of extract (µmol TE/mL of extract).

### 2.9. Oxygen Radical Absorbance Capacity Assay (ORAC)

The ORAC assay was conducted following the procedure established by Dávalos, Gómez-Cordovés, and Bartolomé [35] with some modifications. Firstly, a phosphate buffer (75 mM, pH 7.4) was used, and 20 μL of the sample (after dilution), along with 120 μL of fluorescein (1166.1 µM), was added to a black microplate (Nunc, Denmark). The mixture was pre-incubated at 37 °C for 15 min. After that, 60 μL of 2,2′-azobis-(2-methylpropionamidine)-dihydrochloride (AAPH) (46.6 mM) was added rapidly and then incubated at 37 °C for 140 min. The microplate was read at 1 min intervals using a Multidetection plate reader (Synergy H1, Vermont, USA) at 458 nm and 528 nm. The software used was the Fluostar Control 1.32 R2 version. The calibration curve was made using Trolox (0.0002 to 0.0016 μmol TE/mL) as an antioxidant standard, and the results were expressed in mmol TE/mL of extract. A blank was prepared by using the phosphate buffer instead of a sample. All analyses were carried out in triplicate.

Normalised antioxidant curves were obtained by multiplying the original data by fluorescence_blank,t=0_/fluorescence_sample,t=0_, and dividing by the blank curve corresponding to the same assay. 

### 2.10. α-Glucosidase Inhibition Assay

The α-glucosidase inhibitory activity was measured to assess antidiabetic potential according to the procedure described by Kwon et al. [36], with slight modifications. To begin, 50 μL of the sample was mixed with 100 μL of 0.1 M phosphate buffer (pH = 6.9) containing α-glucosidase solution (1.0 U/mL) in each well. The mixture was then incubated at 25 °C for 10 min. Afterwards, 50 μL of 5 mM *p*-nitrophenyl-a-D-glucopyranoside solution in 0.1 M phosphate buffer (pH = 6.9) was added to each well. Subsequently, the absorbance measurements of the reaction mixtures were taken at 405 nm using a multi-detection plate reader (Synergy H1, VT, USA), after a further 5 min of incubation at 25 °C.

For the experiment, 50 μL of buffer solution was used as the negative control, and 50 μL of acarbose at a concentration of 10 mg/mL was used as the positive control. All assays were performed in triplicate. The inhibition of α-glucosidase was calculated as follows:

$$\alpha -Glucosidase\;inhibition\;(\%)=\left(\frac{\Delta {Abs}_{control}-\Delta {Abs}_{sample}}{\Delta {Abs}_{control}}\right)*100$$

### 2.11. Antibacterial Analysis 

In order to determine the antimicrobial activity of *A. cordata* leaves and roots, as well as the leaves of sea buckthorn and industrial hemp extracts (SLE and EAE_Visc), a well diffusion test method was used based on Kaewchomphunuch et al. [37] with minor modifications. A wide range of pathogenic bacteria were tested, which are listed in Table 2. Each microorganism was grown on Tryptic Soy Agar plates (TSA) at 37 °C for 24 h prior to analysis. The extracts were freeze-dried and then resuspended in 2 mL of water before testing. Agar well diffusion assays were performed in 90 mm diameter Petri dishes containing Mueller Hinton Agar to a depth of 4 ± 0.5 mm. Bacterial suspensions were obtained with a cell concentration of 1 McFarland (around 3 × 10^8^ CFU/mL) in saline solution (0.9% NaCl, Honeywell, Fluka, Cambridge, UK). This suspension was spread uniformly using a sterile cotton swab. Six wells with a 5 mm diameter were used in the agar plates. Then, 50 μL of the test extracts and 10 μL of the positive control, ampicillin, at a concentration of 100 µg/mL was placed into each well using a sterile pipette. The plates were then incubated for 24 h at 37 °C. After incubation, the diameters of the growth inhibition zones were measured in millimetres to the nearest 0.1 mm. Each experiment was repeated three times, and the means and standard deviations were calculated.

### 2.12. Scanning Electron Microscopy (SEM)

This study examined the structure and changes in the samples that were hydrolysed by the Viscozyme L enzyme, using scanning electron microscopy (SEM). The samples, both before and after EAE_Visc, were freeze-dried and mounted on SEM pins using double-sided adhesive carbon tape (NEM tape; Nisshin, Tokyo, Japan). They were then coated with gold/palladium and visualised in a PhenomTM XL G2 (Thermo Fisher Scientific, Eindhoven, The Netherlands) SEM. These analyses were conducted at an accelerating voltage of 5 kV using the secondary electron detector (SED). 

### 2.13. Statistical Analysis

All analyses were performed in triplicate. MS Excel 2020 (Redmond, WA, USA) calculated the mean values and standard deviations. Afterwards, a one-way ANOVA was performed, followed by Tukey’s HSD test to compare the means that showed significant variation (*p* < 0.05). These calculations were performed using GraphPad Prism 8.0 software (GraphPad, San Diego, CA, USA).

## 3. Results and Discussion

### 3.1. Extraction Yield and Concentration

The extraction yields and concentrations of *A. cordata* leaf and root, sea buckthorn leaf, and industrial hemp leaf extracts obtained by solid–liquid extraction (SLE) and enzyme-assisted extraction (EAE) with Viscozyme L (Visc), Cellulase (Cell), and without an enzyme (Control) are presented in Table 3. It can be seen that the extraction methods, as well as the tested plant by-products sources, had significant differences in extraction yield (*p* < 0.05). The extraction of compounds from plant by-products can be challenging due to the complex and diverse polysaccharides present in their cell walls, which can reduce extraction efficiency using classical methods [38]. Degrading the structure of these cell walls is essential to release the compounds. Hydrolytic enzymes were found to increase the extraction yield in plant leaves [39], and this effect was confirmed in the study conducted on all three plant by-products. Regarding the extraction methods and plant by-products, the highest yield was obtained in EAE_Visc extracts in the following order: *A. cordata* leaves, roots, sea buckthorn leaves, and industrial hemp leaves, respectively. 

Meanwhile, there was no significant difference in yield between SLE, EAE_Control, and EAE_Cell in *A. cordata* and industrial hemp leaves (*p* > 0.05, Table 3). However, the highest yield was achieved with EAE_Visc extracts in *A. cordata* leaves, which were 46%, 41%, and 57% higher compared to the SLE, EAE_Control, and EAE_Cell extracts, respectively. In the case of *A. cordata* roots, EAE_Visc extracts delivered a yield 44% higher than EAE_Control extracts. Similarly, the highest yield was obtained in EAE_Visc extracts of industrial hemp leaves and sea buckthorn leaves, which delivered a yield 25%, 44%, and 30% higher in industrial hemp leaves and 46%, 48%, and 77% higher in sea buckthorn leaves compared with the SLE, EAE_Control, and EAE_Cell extracts, respectively. In terms of concentration, a similar correlation can be observed (Table 3). To the best of our knowledge, no previous studies have performed an extraction comparison including the EAE of functional properties from *A. cordata* leaves and roots, sea buckthorn leaves, and industrial hemp leaves. The lack of research on this topic limits the ability to make further comparisons beyond what has been achieved in this study. Generally, these results are compatible with previous reports, indicating that EAE significantly increases the soluble yield in a shorter time frame [40,41].

### 3.2. Characterisation of the Extracts

The composition of total protein, fructose, glucose, sucrose, total sugars, and total phenolics content in different extracts of *A. cordata* leaves and roots, sea buckthorn leaves, and industrial hemp leaves are presented in Table 4. As expected, a higher total protein content was obtained in all tested plant EAE extracts with Viscozyme L and Cellulase enzymes (*p* < 0.05). At the same time, the total protein content did not show a significant difference in the root extract of *A. cordata* in comparison to the other tested plants. Meanwhile, the highest protein content was determined in industrial hemp EAE_Visc extract (11.00 mg/mL of extract), which is almost 2 times higher than EAE_Cell extract and 1.7 times higher than what was obtained in SLE. The total protein content was increasingly higher in sea buckthorn leaves compared with *A. cordata* leaves and roots and industrial hemp leaves. The highest total protein content was detected in sea buckthorn leaves EAE_Cell extract (24.23 mg/mL of extract), 6% higher than in EAE_Visc extract and 30.7% higher than what was obtained in SLE extract. Previous studies have reported that the protein content ranged from 14.90% to 18.60% in sea buckthorn leaf water extract [42], while Ghabru et al. (2023) reported a total protein content of 22.09% in sea buckthorn leaves [43]. Plant cell walls consist of glycoproteins, oligosaccharides, polysaccharides, and other complex carbohydrates that challenge protein extraction [44]. Therefore, enzyme-based technologies allow an increased protein content from plants by degrading the complex carbohydrate-rich plant cell wall. The selection of enzymes is crucial in this process [44,45].

To determine the impact of Viscozyme L, Cellulase, and naturally occurring enzymes on the release and content of simple carbohydrates, the concentrations of mono- and disaccharides in the EAE and SLE extracts were measured using HPLC under optimal conditions. There were no significant differences in total sugar content between the *A. cordata* leaf extracts (*p* > 0.05). Meanwhile, the EAE_Visc extract (10.74 mg/mL of extract) demonstrated a 40% increase in total sugar content compared to the EAE_Control extract (7.77 mg/mL of extract) extracted from *A. cordata* roots. The EAE_Visc (5.05 mg/mL of extract) and EAE_Cell (4.06 mg/mL of extract) extracts of sea buckthorn leaves were shown to have a higher total sugar content, while in the industrial hemp leaves, the EAE_Visc (3.71 mg/mL of extract) and EAE_Control (3.58 mg/mL of extract) showed a higher amount of total sugar content (*p* < 0.05). As expected, the enzyme’s hydrolytic activity significantly increased the saccharide content, particularly glucose and fructose (Table 4). In this case, the results indicate that there was no significant difference between the *A. cordata* leaf extracts, correlating with observations concerning the total sugar content. However, the *A. cordata* root EAE_Visc extract demonstrated a significant increase in glucose and fructose content by 3.8 and 2 times, respectively. Furthermore, a significant difference in glucose value was observed in the EAE_Visc extracts of sea buckthorn and industrial hemp leaves. The sea buckthorn leaves did not show an increase in fructose content, whereas the industrial hemp leaves showed an increase in fructose content in both the EAE_Visc and EAE_Control extracts (*p* < 0.05).

The total phenolic content varied significantly based on the extraction methods and plant sources (*p* < 0.05). Sea buckthorn extracts had the highest total phenolic content, ranging from 200.80 to 285.57 mg/100 mL of extract (Table 4). EAE_Visc extract had significantly the highest value (285.57 mg/100 mL of extract), which was 85%, 75%, and 46% higher compared with the sea buckthorn leaf EAE_Control and EAE_Cell extracts, respectively. A similar tendency was established among the *A. cordata* and industrial hemp leaf extraction methods. Meanwhile, there was no significant difference between the *A. cordata* root extracts. Phenolic compounds in plants exist in soluble and insoluble forms. Soluble phenolics can be extracted easily, while insoluble-bound phenolic compounds are challenging to extract. Insoluble-bound phenolic compounds are covalently bound to plant cell wall structural elements such as cellulose, hemicellulose, structural protein, or polysaccharides [46]. The present study confirmed that using the Viscozyme L cellulolytic enzyme complex resulted in a higher total phenolic content compared to the SLE, EAE_Control, and EAE_Cell methods due to the release of insoluble-bound compounds. The results are consistent with Wang et al. (2017), who conducted a study on enhancing the bioavailability of insoluble-bound phenolics from guava leaves. The study investigated the ability of enzyme-assisted extraction to improve the release of insoluble-bound phenolics and found that complex enzyme-assisted extraction greatly improved the soluble phenolics content [47]. In a study conducted by Habeebullah et al. (2020), the Flavourzyme and Viscozyme L complexes were found to be the most effective enzymes in increasing the total phenolic content of brown seaweed varieties [48].

### 3.3. Quantitative Composition of Phenolic Compounds of Extracts

Phenolic compounds in *A. cordata* leaf and root, sea buckthorn leaf, and industrial hemp leaf extracts were identified and quantified by UHPLC-ESI-MS/MS analysis, as presented in Table 5, Table 6 and Table 7. 

As shown in Table 5, four flavonols and six phenolic acids were identified in *A. cordata* leaf and root extracts. Chlorogenic, *p*-coumaric, and 3,4-dihydroxyphenylacetic acids were found in all tested extracts of *A. cordata* leaves, and neochlorogenic acid was only present in the EAE_Visc extract (Table 5). Neochlorogenic, chlorogenic, ferulic, caffeic, *p*-coumaric, and 3,4-dihydroxyphenylacetic acids were found in all tested extracts of *A. cordata* root. The amount of identified phenolic acids varied significantly (*p* < 0.05). Chlorogenic acid was found to be most abundant in EAE_Visc extract, with the highest amounts of 95.53 µg/mL of extract and 160.23 µg/mL of extract present in *A. cordata* leaves and roots, respectively. In addition, Matsuo et al. [49] also reported studies that *A. cordata* roots have a high concentration of chlorogenic acid, which is essential in regulating glucose and lipid metabolism and managing related disorders such as diabetes, obesity, cardiovascular disease, and cancer [50,51]. 

Regarding the group of flavones, they were only present in *A. cordata* leaves (isoquercitrin, isorhamnetin, isorhamnetin-3-*O*-glucoside, and keampferol-3-*O*-glucoside) (Table 5). Only isorhamnetin was obtained in all extracts, and the amount did not vary significantly. The two most abundant flavonols found in *A. cordata* leaf EAE_Visc extract were isoquercitrin (9.99 µg/mL of extract) and kaempferol-3-*O*-glucoside (8.46 µg/mL of extract). Studies have reported that isoquercitrin can be obtained by the enzymatic hydrolysis of rutin, which, due to its antioxidant activity, scavenges ROS and RNS, including superoxide anion radicals, hydroxyl radicals, peroxyl radicals, and peroxynitrite [52]. In addition, it was reported that kaempferol 3-*O*-glucoside had a hepatoprotective effect on tacrine-induced cytotoxicity in HepG2 cells derived from the human liver [53].

In terms of sea buckthorn leaf extracts, 13 phenolic compounds (4 phenolic acids, 8 flavonols, and 1 flavone) were identified, as shown in Table 6. Ferulic and *p*-coumaric acids were found in all the tested extracts of sea buckthorn leaves, and chlorogenic acid was only obtained in the EAE_Visc extract (Table 6). The largest amount of phenolic compounds was the flavones group in sea buckthorn leaves, including kaempferol-3-*O*-rutinoside, isoquercitrin, isorhamnetin, isorhamnetin-3-*O*-glucoside, isorhamnetin-3-*O*-rutinoside, keampferol-3-*O*-glucoside, rutin, and quercetin. The highest amount was established of gallic acid, isoquercitrin, isorhamnetin-3-*O*-rutinoside, and rutin. Furthermore, luteolin-7-rutinoside was also obtained in all the extracts of sea buckthorn leaves. The phenolic compounds identified in this study align with previously published studies on sea buckthorn leaves [54,55,56,57].

As demonstrated in Table 7, eight phenolic compounds, including four phenolic acids and four flavonols, were identified in industrial hemp leaves. Among these, neochlorogenic, chlorogenic, *p*-coumaric, and 3,4-dihydroxyphenylacetic acids were found only in the EAE_visc extracts of industrial hemp leaves. Additionally, only p-coumaric acid was found in all of the extracts. The amount of identified phenolic acids varied significantly (*p* < 0.05). The most abundant phenolic acid in industrial hemp leaf EAE_Visc extract was chlorogenic acid (91.19 µg/mL of extract), which is similar to that found in *A. cordata* leaves (95.53 µg/mL of extract). However, three flavonols, including isoquercitrin, isorhamnetin-3-*O*-glucoside, and kaempferol-3-*O*-glucoside, were obtained in EAE_Visc, while vitexin-2-rhamnoside was established in the SLE, EAE_Control, and EAE_Cell extracts of industrial hemp leaves.

**Table 7:** Content of phenolic compounds (µg/mL of extract) in industrial leaves extracts.

Phenolic Compound (µg/mL of Extract)	Industrial Hemp Leaves			
	SLE	EAE_Control	EAE_Visc	EAE_Cell
**Phenolic acids**				
Gallic acid	nd	nd	nd	nd
Neochlorogenic acid	nd	nd	0.35 ± 0.03 a	nd
Chlorogenic acid	nd	nd	91.19 ± 0.42 a	nd
Ferulic acid	nd	nd	nd	nd
*p*-coumaric acid	0.33 ± 0.02 a	0.69 ± 0.03 a	2.90 ± 0.30 b	0.46 ± 0.04 a
3,4-dihydroxyphenylacetic acid	nd	nd	0.40 ± 0.41 a	nd
**Flavonols**				
Kaempferol-3-*O*-rutinoside	nd	nd	nd	nd
Isoquercitrin	nd	nd	11.83 ± 0.56 a	nd
Isorhamnetin	nd	nd	nd	nd
Isorhamnetin-3-*O*-glucoside	nd	nd	0.40 ± 0.02 a	nd
Isorhamnetin-3-*O*-rutinoside	nd	nd	nd	nd
Kaempferol-3-*O*-glucoside	nd	nd	8.10 ± 0.54 a	nd
Rutin	nd	nd	nd	nd
Quercetin			nd	nd
Vitexin-2-rhamnoside	14.57 ± 1.52 b	16.63 ± 1.63 b	nd	1.57 ± 0.11 a

nd: not detected. Values are expressed as mean ± standard deviation (*n* = 3); different letters in rows indicate statistically significant differences between extraction methods (one-way ANOVA and Tukey’s HSD test, *p* < 0.05).

### 3.4. Determination of Antioxidant Activity

Plant extracts’ effectiveness as antioxidants depends on their phytochemical composition and bioactive compounds—specifically, the content of phenolic compounds such as flavonoids and phenolic acids [23]. Antioxidants have shown promising results in reducing or halting the progression of various chronic diseases [58]. Plant extracts comprise various biologically active compounds, each possessing its own mechanism of antioxidant activity. This makes it difficult to assess their antioxidant capacity accurately using only one methodology [59]. Multiple methods are recommended to measure the antioxidant capacity of plant extracts accurately [60,61]. Different antioxidant assays (ABTS^•+^, FRAP, and ORAC) were used to evaluate the antioxidant capacity of different sample extracts. The results of antioxidant activity in the tested samples are presented in Table 8. 

The EAE_Visc extracts of the tested plant leaves showed stronger antioxidant activity in all the methods used, whereas no significant differences were found between *A. cordata* root extracts (*p* > 0.05). On the other hand, the EAE_Visc extract of *A. cordata* leaves showed 1.5 and 1.2 times higher antioxidant capacity in ABTS^•+^ compared to the SLE, EAE_Cell, and EAE_Control extracts, respectively. The ABTS^•+^ antioxidant activity in vitro of industrial hemp extracts varied similarly (*p* < 0.05). Meanwhile, the EAE_Visc extract (46.11 µmol TE/mL of extract) of sea buckthorn leaves was reported to have the highest antioxidant capacity in ABTS^•+^ compared with the other investigated plants. For the ferric reducing antioxidant power (FRAP), the EAE_Visc extracts were capable of a 46–50% and 18–62% increase in scavenging activity in *A. cordata* and sea buckthorn leaves, respectively (Table 8). Meanwhile, the FRAP-reducing activity in vitro in the EAE_Visc extracts of industrial hemp leaves was 1.6–4.2 times higher than in the other tested extracts. Furthermore, the antioxidant capacities obtained with the oxygen radical absorbance capacity (ORAC) assay showed a similar correlation with the ABTS^•+^ and FRAP in the tested extracts. However, the tested extracts obtained higher values when measured by the ORAC method. This can be explained by the difference in the working mechanisms, where the ORAC method uses the hydrogen atom transfer radical-quenching mechanism of peroxyl radicals [62]. 

### 3.5. Antidiabetic Properties

**Figure 2:**
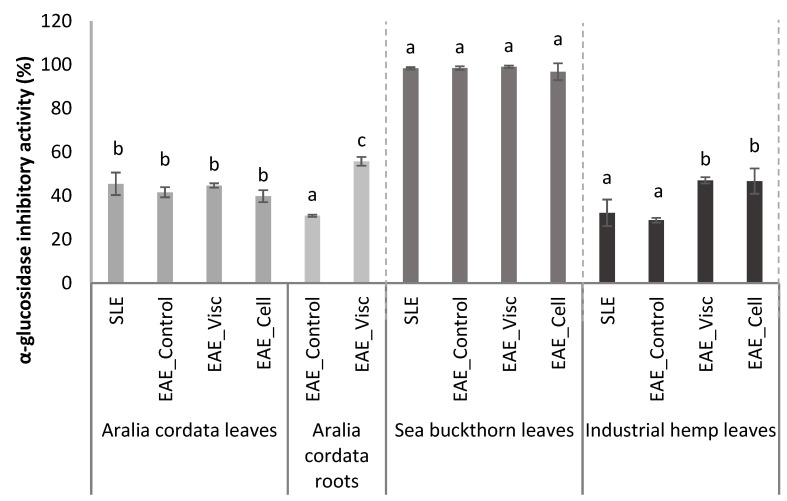
α-glucosidase inhibitory activity of *A. cordata* leaves and roots, sea buckthorn leaves, and industrial hemp leaves for all analysed conditions. Values are expressed as mean ± standard deviation (*n* = 3); different letters indicate statistically significant differences between extraction methods (one-way ANOVA and Tukey’s HSD test, *p* < 0.05).

Type 2 diabetes is a significant public health concern with a growing burden worldwide [63]. It affects quality of life and contributes to substantial morbidity and mortality, especially in younger people [64]. Unhealthy lifestyles and an aging population are the leading causes [65]. Additionally, Chen et al. (2020) [66] and Amaliah et al. (2019) [67] in their studies demonstrated the blood sugar-lowering effects of *Moringa oleifera* and Siam weed leaves, respectively. The obtained antidiabetic activity of different extracts of *A. cordata* leaves and roots, sea buckthorn leaves, and industrial hemp leaves are presented in Figure 2. 

The inhibitory activity of α-glucosidase in extracts from the leaves and roots of *A. cordata* varied between 41.61% to 45.50% and 30.87% to 55.78%, respectively. The extraction method used for *A. cordata* leaves did not show any significant differences. However, there were significant differences between the extraction methods used for *A. cordata* roots (*p* < 0.05). The EAE_Visc extract in *A. cordata* roots showed a 25% higher α-glucosidase inhibitory activity compared to the EAE_Control extract. On the other hand, the α-glucosidase inhibitory activity in different industrial hemp extracts ranged from 28.8% to 47.1%. Statistical differences were observed between the SLE and EAE_Control extracts, and the EAE_Visc and EAE_Cell extracts, which indicates that enzyme-assisted extraction has a positive effect on increasing α-glucosidase inhibitory activity in industrial hemp leaves (*p* < 0.05, Figure 2). However, no significant difference between sea buckthorn leaf extracts was indicated. Nevertheless, the highest α-glucosidase inhibitory activity was obtained in sea buckthorn leaves, almost reaching 99% in all extracts (Figure 2). These results are consistent with previous studies demonstrating sea buckthorn leaves’ significant antidiabetic potential [14]. Research by Bhardwaj et al. (2015) [68] has shown that the methanolic extract of these leaves possesses a potent inhibitory action against α-glucosidase—a crucial enzyme in carbohydrate metabolism. This was further supported by Sharma et al. (2011) [69], who found that the leaves can reduce blood glucose levels and oxidative stress in diabetic rats. These findings collectively suggest that sea buckthorn leaves could be a natural antidiabetic agent.

### 3.6. Antibacterial Properties

It was previously reported that sea buckthorn leaves possess antimicrobial activity against various pathogens and can be promising antimicrobial alternatives in the food, pharmacy, and cosmetic industries [70]. In this study, the antimicrobial activity of *A. cordata* leaf and root, sea buckthorn leaf, and industrial hemp leaf SLE and EAE_Visc extracts were investigated against seven pathogens: *Streptococcus intermedius*, *Klebsiella pneumoniae*, *Escherichia coli*, *Staphylococcus aureus*, *Salmonella enterica*, *Yersinia enterocolitica*, and *Listeria monocytogenes*. However, *A. cordata* leaves and roots, as well as the industrial hemp leaf tested extracts, did not show inhibition effects against all the tested pathogenic bacteria. Therefore, these results are not presented in Table 9. On the other hand, the water-based SLE and EAE_Visc sea buckthorn extracts showed positive effects against Gram-positive *Staphylococcus aureus* (*S. aureus*) bacteria, as presented in Table 9. 

Upadhyay et al. reported a study on the antibacterial activity of sea buckthorn leaf water and 70% ethanol extracts and found that, depending on the concentration, water-based extracts can positively inhibit *S. aureus* and *E. coli* pathogens [70]. Another study conducted by Criste et al. confirms the antibacterial activity of sea buckthorn leaves against *S. aureus*, but it also highlights that the effectiveness of the inhibition may vary depending on the type of plant species [11]. Different extraction methods and solvents can result in variations in the types and quantities of compounds extracted, thereby influencing their antimicrobial activity. Overall, these studies demonstrate that sea buckthorn leaves can be a valuable natural source of antibacterial agents.

### 3.7. Scanning Electron Microscopy Analysis of Plant Material before and after EAE

**Figure 3:**
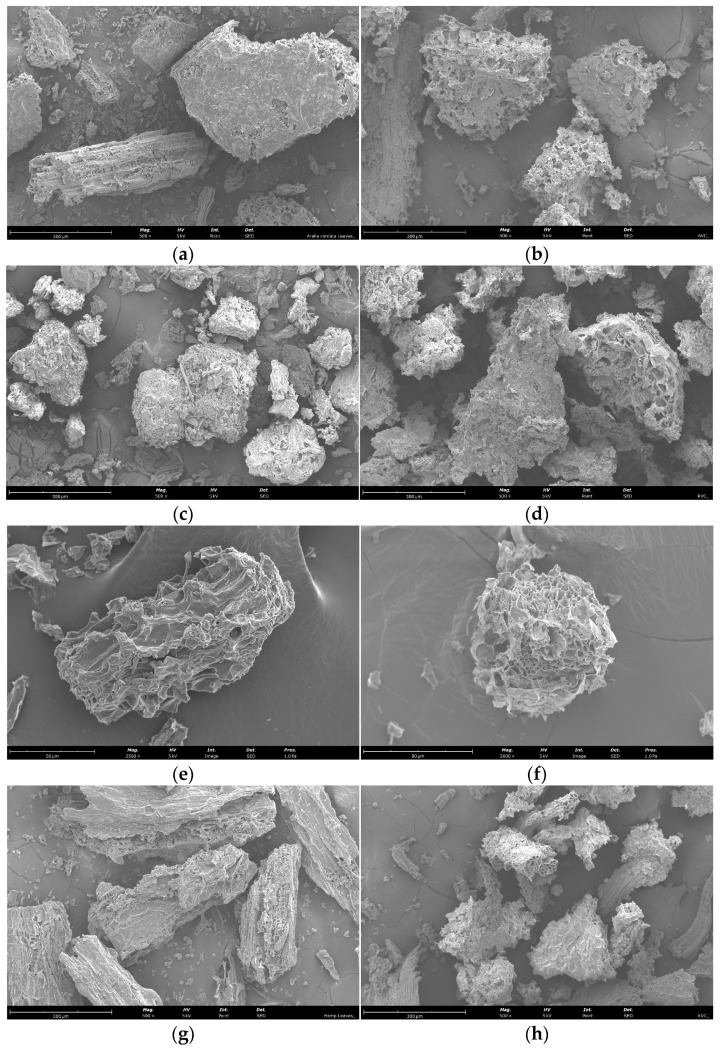
SEM micrographs of *A. cordata* leaves (**a**,**b**) and roots (**c**,**d**), sea buckthorn leaves (**e**,**f**), and industrial hemp leaves (**g**,**h**) before (**a**,**c**,**e**,**g**) and after EAE (**b**,**d**,**f**,**h**), respectively. The images are at 500×, 2500× and 2000× magnification, and scale bars represent 300, 50 and 80 µm ((**a**–**h**), respectively).

The impact of EAE on cell wall degradation was assessed using scanning electron microscopy. Figure 3 depicts the microstructure of the plant material before and after treatment with the Viscozyme L enzyme. The control sample (Figure 3a,c,e,g) showed a smooth surface without any ruptures or significant disruption to the microstructure. However, after using Viscozyme L, a considerable amount of tissue fragments were noticed (see Figure 3b,d,f,h) that were coated with various small particles. Furthermore, partial exfoliation and changes in morphology were detected on the cell surface. After hydrolysis, the surface appeared rough and uneven and was more susceptible to destruction. In Figure 3b,d,f,h, the process of hydrolysis and the resulting changes in the shape of individual particles can be observed. Based on these observations, the alterations in morphology are a result of the hydrolysis process. Our prior research on sea buckthorn leaves had revealed consistent morphological alterations in cell walls exposed after EAE with the Viscozyme L enzyme [8].

The structural support and protection of the plant cell wall depends on its complex composition that prevents the release of intracellular components [71,72]. It is possible for bioactive compounds to exist in both bound and free states. The extraction of these compounds can be significantly improved by breaking down the cell wall through enzymatic degradation [73]. This process results in a significantly higher yield of these compounds and enhances their antioxidant activity [74,75]. This fact is supported by the changes in morphology that can be observed (Figure 3). Accordingly, the prior research has revealed that enzymatic hydrolysis results in discernible modifications to cell wall morphology [76,77].

## 4. Conclusions

This study has shown that the leaves and roots of *A. cordata,* and sea buckthorn and industrial hemp leaves, can be a natural and cost-effective source of bioactive compounds. This might be also extendable to other agro-industrial by-products, with various potential applications in different industries. Enzyme-assisted extraction is proposed as a promising method for obtaining extracts with engaging biological properties, especially using the Viscozyme L enzyme. In this study, the EAE_Visc extracts from the leaves of *A. cordata*, sea buckthorn, and industrial hemp showed the highest yield, antioxidant activity, and total phenolic content. In the meantime, the EAE_Visc extract for *A. cordata* roots possesses a significant amount of sugars. The most abundant and the highest amount of chlorogenic acid was obtained in the EAE_Visc extracts of industrial hemp (91.19 µg/mL of extract) and *A. cordata* leaves (95.53 µg/mL of extract). Nevertheless, the sea buckthorn leaves’ α-glucosidase inhibitory activity was reported and almost reached 99% in all extracts, which shows an attractive antidiabetic potential. Moreover, sea buckthorn leaf SLE and EAE_Visc extracts possess antibacterial activity against *Staphylococcus aureus*. Furthermore, SEM images of the tested plants identified successful cleavage and hydrolysis by hydrolytic enzymes. In general, enzyme-assisted extraction is an effective method for extracting high-yield bioactive compounds by selecting specific enzymes and is an excellent alternative to conventional extraction methods.

## Figures and Tables

**Table 1:** Parameters of mass spectrometry for analysis of phenolic compounds.

Compound	Parent Ion (*m*/*z*)	Daughter Ion (*m*/*z*)	Cone Voltage, V	Collision Energy, eV
3,4-dihydroxyphenylacetic acid	153	81	32	20
*p*-coumaric acid	163	93	28	22
Gallic acid	169	51	36	30
Caffeic acid	179	107	30	20
Ferulic acid	193	134	32	18
Quercetin	301	151	48	20
Isorhamnetin	315	300	44	22
Neochlorogenic acid	353	191	32	14
Chlorogenic acid	353	191	32	14
Kaempherol-3-*O*-glucoside	447	284	54	28
Isoquercitrin	463	301	52	28
Hyperoside	463	300	50	26
Isorhamnetin-3-glucoside	477	314	60	28
Vitexin-2-rhamnoside	578	293	64	36
Kaempferol-3-*O*-rutinoside	593	285	36	20
Luteolin-7-rutinoside	593	285	82	36
Rutin	609	300	70	38
Isorhamnetin-3-*O*-rutinoside	623	315	70	32

**Table 2:** Targeted microorganisms and their sources used for antimicrobial analysis.

Pathogens	Source
*Streptococcus intermedius* 2567	ESB culture collection
*Klebsiella pneumoniae* ESB	
*Escherichia coli* ATCC 25922	ATCC
*Staphylococcus aureus* ATCC 25923	
*Salmonella enterica* serovar *thyphimurium* ATCC 14028	
*Yersinia enterocolitica* NCTC 10460	NCTC
*Listeria monocytogenes* NCTC 10357	

ESB—Escola Superior de Biotecnologia; ATCC—American Type Culture Collection; NCTC—National Collection of Types cultures.

**Table 3:** Extraction yields and concentrations of tested sample extracts.

Samples	Extraction Method	Concentration	Yield
		g/mL	g/100g DW
*Aralia cordata* leaves	SLE	0.018 ± 0.001 a	23.04 ± 1.18 a
	EAE_Control	0.019 ± 0.002 a,b	23.84 ± 0.83 a
	EAE_Visc	0.024 ± 0.002 b	33.61 ± 3.68 b
	EAE_Cell	0.014 ± 0.001 a	21.43 ± 1.98 a
*Aralia cordata* roots	EAE_Control	0.018 ± 0.001 a	22.29 ± 0.53 a
	EAE_Visc	0.025 ± 0.003 b	31.98 ± 2.50 b
Sea buckthorn leaves	SLE	0.015 ± 0.001 a	18.15 ± 1.08 b
	EAE_Control	0.015 ± 0.001 a	17.79 ± 1.31 b
	EAE_Visc	0.021 ± 0.001 b	26.41 ± 0.56 c
	EAE_Cell	0.012 ± 0.001 a	14.93 ± 0.55 a
Industrial hemp leaves	SLE	0.014 ± 0.002 a,b	17.38 ± 2.41 a
	EAE_Control	0.012 ± 0.001 a,b	15.04 ± 0.76 a
	EAE_Visc	0.015 ± 0.001 b	21.70 ± 0.96 b
	EAE_Cell	0.012 ± 0.001 a	16.70 ± 1.59 a

Values are expressed as mean ± standard deviation (*n* = 3); different letters in columns indicate statistically significant differences between extraction methods (one-way ANOVA and Tukey’s HSD test, *p* < 0.05).

**Table 4:** Contents of protein, sucrose, glucose, fructose, total sugars, and total phenolics in the different extracts of *A. cordata* leaves and roots, sea buckthorn leaves, and industrial hemp leaves.

Samples	Extraction Method	Protein	Sucrose	Glucose	Fructose	Total Sugars	Total Phenolics Content
		mg/mL of extract	mg/mL of extract	mg/mL of extract	mg/mL of extract	mg/mL of extract	mg GAE/100 mL of extract
*Aralia cordata* leaves	SLE	6.68 ± 0.047 b	0.29 ± 0.004 a	0.95 ± 0.031 a	1.93 ± 0.054 b	3.18 ± 0.019 a	81.40 ± 8.171 b
	EAE_Control	9.84 ± 0.072 c	0.29 ± 0.077 a	1.17 ± 0.115 a	2.18 ± 0.087 b	3.54 ± 0.125 a	88.00 ± 1.682 b
	EAE_Visc	9.80 ± 0.156 c	0.21 ± 0.034 a	1.07 ± 0.135 a	2.26 ± 0.011 b	3.64 ± 0.158 a	104.30 ± 6.817 c
	EAE_Cell	10.28 ± 0.060 d	0.28 ± 0.004 a	1.28 ± 0.151 a	2.05 ± 0.015 b	3.49 ± 0.170 a	93.00 ± 5.444 b,c
*Aralia cordata* roots	EAE_Control	4.62 ± 0.073 a	4.79 ± 0.133 c	1.46 ± 0.022 a	1.41 ± 0.143 a	7.66 ± 0.298 b	40.45 ± 1.061 a
	EAE_Visc	4.44 ± 0.050 a	2.20 ± 0.159 b	5.66 ± 0.654 b	2.88 ± 0.185 c	10.74 ± 0.311 c	36.10 ± 1.414 a
Sea buckthorn leaves	SLE	18.54 ± 0.154 b	0.54 ± 0.043 a	1.28 ± 0.010 b,c	1.98 ± 0.102 a	3.80 ± 0.135 a,b	200.80 ± 4.812 a
	EAE_Control	17.37 ± 0.364 a	0.54 ± 0.111 a	0.83 ± 0.096 a	1.71 ± 0.103 a	3.08 ± 0.117 a	210.70 ± 8.581 a
	EAE_Visc	22.84 ± 0.107 c	1.57 ± 0.069 c	1.41 ± 0.269 c	2.06 ± 0.278 a	5.03 ± 0.616 b	285.57 ± 6.367 c
	EAE_Cell	24.23 ± 0.601 d	1.11 ± 0.086 b	1.12 ± 0.094 a,b	1.82 ± 0.067 a	4.06 ± 0.075 a,b	239.70 ± 7.873 b
Industrial hemp leaves	SLE	6.52 ± 0.058 b	0.39 ± 0.081 a	0.68 ± 0.010 a	0.81 ± 0.117 a	1.88 ± 0.189 a	68.17 ± 2.250 b
	EAE_Control	9.72 ± 0.151 c	0.32 ± 0.021 a	1.07 ± 0.038 b,c	2.20 ± 0.080 b	3.58 ± 0.020 b	78.17 ± 7.282 b
	EAE_Visc	11.00 ± 0.227 d	0.22 ± 0.030 a	1.31 ± 0.103 c	2.18 ± 0.237 b	3.71 ± 0.163 b	106.07 ± 7.862 c
	EAE_Cell	5.93 ± 0.131 a	0.30 ± 0013 a	0.90 ± 0.061 a,b	1.13 ± 0.111 a	2.32 ± 0.64 a	51.73 ± 2.230 a

Values are expressed as mean ± standard deviation (*n* = 3); different letters in columns indicate statistically significant differences between extraction methods (one-way ANOVA and Tukey’s HSD test, *p* < 0.05).

**Table 5:** Nature and content of phenolic compounds (µg/mL of extract) in *A. cordata* leaves and root extracts.

Phenolic Compound, µg/mL of Extract	*Aralia cordata* Leaves				*Aralia cordata* Roots	
	SLE	EAE_Control	EAE_Visc	EAE_Cell	EAE_Control	EAE_Visc
**Phenolic acids**						
Neochlorogenic acid	nd	nd	0.41 ± 0.11 a	nd	6.21 ± 0.65 b	7.09 ± 0.91 b
Chlorogenic acid	7.39 ± 0.34 a,b	0.57 ± 0.07 a	95.53 ± 2.74 c	17.91 ± 0.07 b	151.15 ± 6.06 d	160.23 ± 3.28 d
Ferulic acid	nd	nd	nd	nd	0.14 ± 0.01 a	0.15 ± 0.01 a
Caffeic acid	nd	nd	nd	nd	1.30 ± 0.14 a	2.36 ± 0.01 b
*p*-coumaric	0.20 ± 0.01 a	0.32 ± 0.05 a,b	2.58 ± 0.06 d	0.33 ± 0.01 a,b	0.47 ± 0.06 b	0.77 ± 0.12 c
3,4-dihydroxyphenylacetic acid	0.29 ± 0.02 a	nd	0.16 ± 0.02 a	0.18 ± 0.01 a	6.31 ± 0.24 b	7.26 ± 0.09 c
**Flavonols**						
Isoquercitrin	nd	nd	9.99 ± 0.77 a	nd	nd	nd
Isorhamnetin	0.27 ± 0.02 a	0.52 ± 0.05 a	0.39 ± 0.02 a	0.16 ± 0.01 a	nd	nd
Isorhamnetin-3-*O*-glucoside	nd	nd	0.40 ± 0.09 a	nd	nd	nd
Kaempferol-3-*O*-glucoside	nd	nd	8.46 ± 0.21 a	nd	nd	nd

nd: not detected. Values are expressed as mean ± standard deviation (*n* = 3); different letters in rows indicate statistically significant differences between extraction methods (one-way ANOVA and Tukey’s HSD test, *p* < 0.05).

**Table 6:** Content of phenolic compounds (µg/mL of extract) in sea buckthorn leaves extracts.

Phenolic Compound (µg/mL of Extract)	Sea Buckthorn Leaves			
	SLE	EAE_Control	EAE_Visc	EAE_Cell
**Phenolic acids**				
Gallic acid	11.94 ± 0.18 a	14.69 ± 0.03 b	12.04 ± 0.13 a	22.58 ± 0.08 c
Neochlorogenic acid	nd	nd	nd	nd
Chlorogenic acid	nd	nd	0.12 ± 0.01 a	nd
Ferulic acid	0.67 ± 0.04 a	1.10 ± 0.15 a	0.40 ± 0.05 a	0.54 ± 0.08 a
*p*-coumaric acid	0.35 ± 0.05 a	0.37 ± 0.03 a	0.45 ± 0.04 a	0.41 ± 0.05 a
3,4-dihydroxyphenylacetic acid	nd	nd	nd	nd
**Flavonols**				
Kaempferol-3-*O*-rutinoside	0.16 ± 0.03 a	0.18 ± 0.06 a	0.16 ± 0.01 a	0.39 ± 0.01 b
Isoquercitrin	2.43 ± 0.16 a	4.39 ± 0.22 b	5.64 ± 0.36 c	4.26 ± 0.01 b
Isorhamnetin	0.22 ± 0.02 a	0.54 ± 0.05 b	0.31 ± 0.02 a	0.93 ± 0.08 c
Isorhamnetin-3-*O*-glucoside	1.16 ± 0.01 a	2.43 ± 0.16 a,b	3.39 ± 0.04 b	2.23 ± 0.07 a,b
Isorhamnetin-3-*O*-rutinoside	4.88 ± 0.13 b	5.58 ± 0.48 b,c	3.57 ± 0.13 a	6.45 ± 0.32 c
Kaempferol-3-*O*-glucoside	0.34 ± 0.06 a	1.06 ± 0.10 b	1.18 ± 0.06 b	1.04 ± 0.13 b
Rutin	6.29 ± 0.23 a,b	6.51 ± 1.00 a,b	4.86 ± 0.15 a	7.65 ± 0.09 b
Quercetin	nd	0.13 ± 0.02 a	0.12 ± 0.01 a	0.26 ± 0.17 a
Vitexin-2-rhamnoside	nd	nd	nd	nd
**Flavones**				
Luteolin-7-rutinoside	0.19 ± 0.02 a,b	0.18 ± 0.02 a,b	0.13 ± 0.02 a	0.42 ± 0.07 b

nd: not detected. Values are expressed as mean ± standard deviation (*n* = 3); different letters in rows indicate statistically significant differences between extraction methods (one-way ANOVA and Tukey’s HSD test, *p* < 0.05).

**Table 8:** Analysis of antioxidant activity in different extracts of *A. cordata* leaves and roots, sea buckthorn leaves, and industrial hemp leaves using ABTS^•+^, FRAP, and ORAC assays.

Samples	Extraction Method	ABTS^•+^	FRAP	ORAC
		µmol TE/mL of extract	µmol TE/mL of extract	mmol TE/mL of extract
*Aralia cordata* leaves	SLE	6.77 ± 0.34 b	2.80 ± 0.19 b	13.16 ± 0.68 b
	EAE_Control	8.59 ± 0.48 c	2.75 ± 0.17 a,b	15.65 ± 0.57 b,c
	EAE_Visc	10.36 ± 0.41 d	4.12 ± 0.39 c	21.71 ± 1.84 d
	EAE_Cell	6.93 ± 0.21 b	2.83 ± 0.26 b	18.35 ± 1.58 c,d
*Aralia cordata* roots	EAE_Control	2.88 ± 0.50 a	1.49 ± 0.08 a	6.77 ± 0.38 a
	EAE_Visc	2.95 ± 0.15 a	1.89 ± 0.59 a	5.26 ± 0.24 a
Sea buckthorn leaves	SLE	32.51 ± 0.47 a	18.36 ± 0.47 a	14.13 ± 2.13 a,b
	EAE_Control	36.26 ± 0.59 b	21.40 ± 0.56 b	11.99 ± 0.58 a
	EAE_Visc	46.11 ± 0.59 d	29.85 ± 0.45 d	20.38 ± 0.51 c
	EAE_Cell	39.42 ± 1.01 c	25.31 ± 0.59 c	17.65 ± 1.25 b,c
Industrial hemp leaves	SLE	6.57 ± 0.31 b	1.12 ± 0.44 a	7.63 ± 0.64 a
	EAE_Control	8.07 ± 0.46 c	2.94 ± 0.43 b	17.23 ± 1.12 b
	EAE_Visc	10.17 ± 0.45 d	4.67 ± 0.49 c	22.54 ± 2.55 c
	EAE_Cell	5.22 ± 0.43 a	2.87 ± 0.13 b	6.82 ± 0.37 a

Values are expressed as mean ± standard deviation (*n* = 3); different letters indicate statistically significant differences between extraction methods (one-way ANOVA and Tukey’s HSD test, *p* < 0.05).

**Table 9:** Antibacterial activity determination of sea buckthorn leaf extract.

Pathogens	Inhibition Zone, mm	
	SLE	EAE_Visc
*Streptococcus intermedius*	-	-
*Klebsiella pneumoniae*	-	-
*Escherichia coli*	-	-
*Staphylococcus aureus*	2.33 ± 0.58 a	4.17 ± 0.29 b
*Salmonella enterica*	-	-
*Yersinia enterocolitica*	-	-
*Listeria monocytogenes*	-	-

(-) indicates no detected inhibition zones for tested concentration. Values are expressed as mean ± standard deviation (*n* = 3); different letters indicate statistically significant differences between extraction methods (one-way ANOVA and Tukey’s HSD test, *p* < 0.05).

## Data Availability

All data generated during this study are included in this article.
